# Measurement of Scattering Cross Section with a Spectrophotometer with an Integrating Sphere Detector

**DOI:** 10.6028/jres.117.012

**Published:** 2012-09-13

**Authors:** A. K. Gaigalas, Lili Wang, V. Karpiak, Yu-Zhong Zhang, Steven Choquette

**Affiliations:** 1 National Institute of Standards and Technology, Gaithersburg, MD 20899; 2 Life Technologies, 29851 Willow Creek Rd., Eugene, OR 97402

**Keywords:** integrating sphere detector, Lorenz-Mie scattering, microspheres, scattering

## Abstract

A commercial spectrometer with an integrating sphere (IS) detector was used to measure the scattering cross section of microspheres. Analysis of the measurement process showed that two measurements of the absorbance, one with the cuvette placed in the normal spectrometer position, and the second with the cuvette placed inside the IS, provided enough information to separate the contributions from scattering and molecular absorption. Measurements were carried out with microspheres with different diameters. The data was fitted with a model consisting of the difference of two terms. The first term was the Lorenz-Mie (L-M) cross section which modeled the total absorbance due to scattering. The second term was the integral of the L-M differential cross section over the detector acceptance angle. The second term estimated the amount of forward scattered light that entered the detector. A wavelength dependent index of refraction was used in the model. The agreement between the model and the data was good between 300 nm and 800 nm. The fits provided values for the microsphere diameter, the concentration, and the wavelength dependent index of refraction. For wavelengths less than 300 nm, the scattering cross section had significant spectral structure which was inversely related to the molecular absorption. This work addresses the measurement and interpretation of the scattering cross section for wavelengths between 300 nm and 800 nm.

## 1. Introduction

The measurement of absorption and scattering from micro particles is an important problem. Such measurements are used to characterize impurities in water [1], develop designs for bio threat detectors, and characterize aquatic particles [2]. A spectrophotometer which measures the decrease in the intensity of an incident collimated light beam can be used to make absorbance measurements. The decrease in the intensity of the incident light can be due to molecular absorption or the scattering of light out of the incident beam. In either case the spectrophotometer records an absorbance. A major problem in measuring the absorbance due to scattering is the finite aperture of the detector for forward scattered light. The spectrophotometer with an integrating sphere (IS) detector can be used to change the relative amount of scattered light which enters the detector. The IS with the sample cuvette holder inside accepts all light leaving the cuvette; it provides the least sensitivity to scatted light and greatest sensitivity to molecular absorption. The conventionally located sample cuvette holder outside the IS detector yields a response which is sensitive to both the loss due to scattering and the loss due to molecular absorption. By combining the measurements in the two holders it is possible to separate the two sources of absorbance. The loss due to scattering can be related to the scattering cross section which, in the case of homogeneous spheres, is given by Lorenz- Mie (L-M) theory. The validity of the L-M theory has been demonstrated many times [3,4]. Recent measurements by He et. al. of the scattering cross section of microspheres [5] used a special spectrophotometer with an extremely small acceptance aperture to minimize the biases due to forward scattered light entering the detector. He’s measurements showed that the L-M theory gives a good description of scattering of light from microspheres. Therefore it is reasonable to assume that the L-M scattering theory is valid, and to examine the possibility of using a conventional spectrometer to measure the properties of microsphere suspensions. In this work we use a commercial spectrophotometer and include a finite acceptance aperture of the detector as a parameter in the interpretation of the measurements. The results suggest that the additional parameter (finite acceptance aperture) is sufficient to obtain a good fit between measurements and the predictions of L-M theory. Consequently a commercial spectrophotometer with an IS detector could be used to determine microsphere diameter, the concentration of microsphere suspension, and the wavelength dependence of the index of refraction of the microsphere material. This work applies the measurement model developed previously [6] to the measurement and interpretation of the scattering cross section of microspheres suspended in an aqueous solution.

## 2. Interpretation of Integrating Sphere (IS) Measurements

**Fig. 1:**
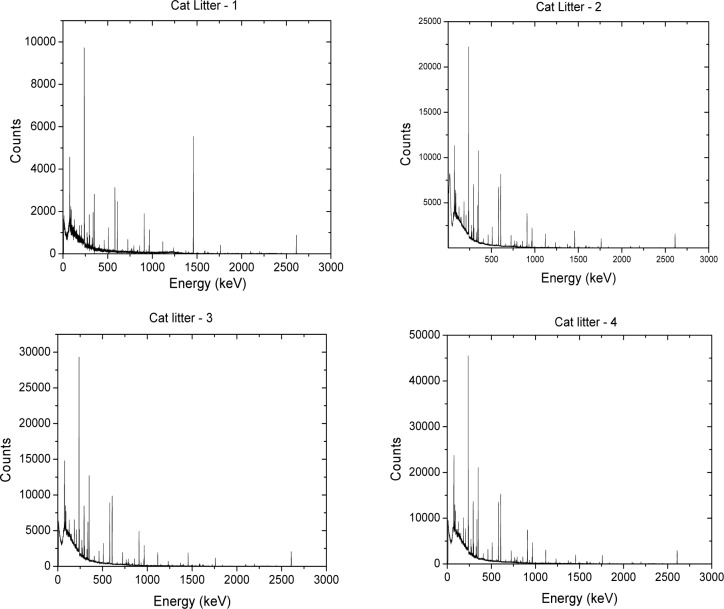
A schematic of the Perkin Elmer dual beam Lambda 850 spectrophotometer sample holders. Holder 1 represents the normal cuvette holder. Holder 2 locates the cuvette in front of the entrance port of the integrating sphere (IS) detector. Holder 3 places the cuvette inside the IS detector. For all cuvette positions, the same reference beam enters the IS detector through a reference port and hits the wall of the IS detector. In practice, the same ‘auto zero’ is used for all cuvette holders. The reference beam also has a holder which is not shown in the diagram.

The proposed method for measuring the scattering cross section of microspheres utilizes a commercial spectrophotometer with an integrating sphere (IS) detector. The method was described previously [6] and here we provide only a short summary. The path of the light beam that probes the sample contains three mechanical cuvette holders labeled 1, 2, and 3 in Fig. 1. Two cuvette holders (1 and 2) are located outside of the IS detector while the third cuvette holder (3) is located inside the IS detector. The mirrors shape the light beam so that it passes unobstructed through the cuvette holders. Two measurements of absorption can be performed with the cuvette placed outside the IS, and the third measurement with the cuvette inside the IS. The response in holder 3 is complicated by the detection of fluorescence and the settling of suspensions due to inability to stir in holder 3.

**Fig. 2:**
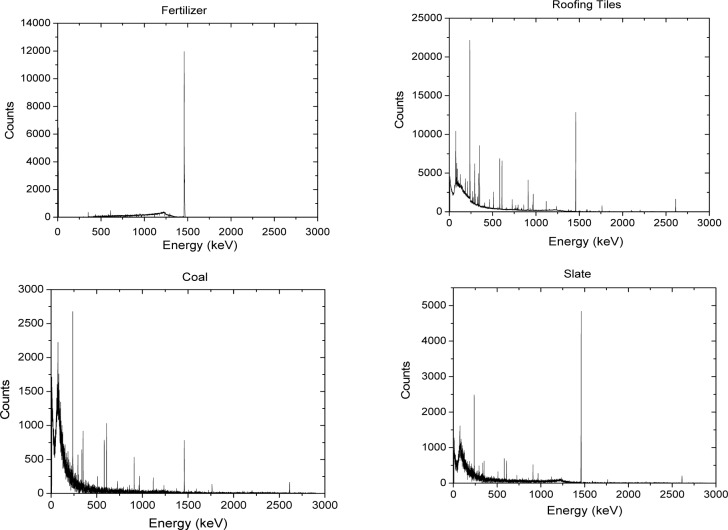
A model of the light fluxes present in the cuvette. The incident flux Φ*_i_* is attenuated by the passage through the cuvette and exits the back of the cuvette as a flux Φ*_t_*. Along the path of the incident flux, a scatter flux arises due to scattering from particles in the suspension. The scattered flux, Φ*_s_*, exits the cuvette and some of the scattered flux may enter the IS detector.

Figure 2 shows a model of the expected response when a beam of light passes through a cuvette filled with the microsphere suspension. The two vertical lines in Fig. 2 represent the walls of the cuvette and the horizontal arrows represent the incident and transmitted light beam. The total absorbance coefficient, *a* =*σ N*, is expressed as a product of the total absorbance cross section, *σ*, in units of cm^2^ and the concentration of microspheres, *N*, in units of cm^−3^. The path length through the cuvette is 1 cm. The total absorbance coefficient will be written as *a* = *a_s_ a_m_*+ where *a_s_* =*σ_s_N* is the apparent absorption coefficient due to scattering, and *a_m_* =*σ_m_N* is the molecular absorption coefficient. This description assumes that a scattered photon is not detected and therefore appears to have been absorbed. The effect of the first cuvette wall is represented by a change in the incident photon flux, Φ*_i_* to *t*Φ*_i_* = 10^log^*^t^* Φ*_i_* where *t* is the transmission of the cuvette wall. The differential equation can be solved to find the flux after the beam has traversed the fluid inside the cuvette. The result is *t*Φ *e*^−^*^al^* =*t*Φ*_i_* 10^−0.434^*^al^* where *l* is the path length which will be set to 1 cm. The second cuvette wall introduces another transmission coefficient *t* giving the final transmitted flux Φ*_t_* =*t*^2^Φ*_i_*10^−0.434^*^al^* Next, consider the scattered flux which originates from the scattering of the incident flux by the microspheres inside the cuvette. Some of the scattered flux will be in the forward direction and thus it will enter the detector since most detectors have a finite acceptance aperture. The scattered flux, which enters the detector, can be estimated by solving the model differential equations shown in Fig 2. The result is given in Eq. (1).

(1) $$\Phi_{s}=t\frac{a_{sp}}{a_{s}+a_{m}}(1-10^{-0.434(a_{s}+a_{m})})t\Phi_{i}$$

Where the partial scattering cross section is given by Eq. (2) [7].

(2) $$\begin{array}{c} \sigma_{sp}=\frac{\pi r^{2}}{x^{2}}\int_{0}^{\Delta }\left(|S_{1}(\theta ){|}^{2}+S_{2}(\theta ){|}^{2}\right)\sin(\theta )d\theta \\ x=\frac{2\cdot \pi \cdot r}{\lambda }\cdot n_{medium} \end{array}$$

Where *r* stands for the radius of the microsphere and the quantities *S*_1_(*θ*) and *S*_2_(*θ*) were calculated using Maetzler code **Mie_S12** [8]. The total scattering and absorption cross sections*σ_s_* and *σ_m_* were obtained from Maetzler code **mie**(m,x**)** where m stands for the ratio of the index or refraction of the microsphere material and the index of refraction of the medium. The Maetzler code reflects the formalism presented by Bohren and Huffman [4]. The quantity *σ_sp_* gives the flux scattered into an angle Δ which in practice is related to the detector acceptance aperture as seen from the location of the cuvette. Equation (1) assumes that there is no additional attenuation of the scattered photon flux, and that multiple scattering is not important. Therefore the expression in Eq. (1) is an approximation which is valid for dilute suspensions and suspensions with small molecular absorption. Finally the transmitted and the scattered fluxes can be written in terms of the incident flux and the properties of the cuvette to give the apparent absorbance, A, registered by the instrument as shown in Eq. (3).

(3) $$10^{-A}=t^{2}10^{-0.434(a_{s}+a_{m})}+t^{2}\frac{a_{sp}}{a_{s}+a_{m}}(1-10^{-0.434(a_{s}+a_{m})})$$

The two terms in Eq. (3) represent the contributions from the transmitted flux and the scattered flux respectively. The value of observed absorbance, *A*, can be compared directly to the function given by the model in Eq. (3). If the cuvette is filled with a buffer which has no scattering (*a_s_* = 0) and no molecular absorption (*a_m_* = 0) then Eq. (3) suggests that the transmission coefficient through the cuvette walls can be evaluated using Eq. (4).

(4) $$t^{2}=10^{2\log t}=10^{-A_{buf}}$$

A measurement of the absorption with buffer in the cuvette gives the absorption due to the finite transmittance at the cuvette walls, which can be used to estimate the *t* ^2^ factor in Eq. (3). Multiplying both sides of Eq. (3) with $10^{A_{buf}}$ yields the final expression for the measurement model with blank correction.

(5) $$10^{-(A-A_{buf})}=10^{-0.434(a_{s}+a_{m})}+\frac{a_{sp}}{a_{s}+a_{m}}(1-10^{-0.434(a_{s}+a_{m})})$$

The relation in Eq. (5) is applicable to measurements performed in any of the three holders. The measured absorbance should be very different for samples placed in cuvette holder 1, 2, and 3 since the detector acceptance angle is very different for the three holders.

In many applications the measured total absorbance is less than 0.2. In that case the analysis is simplified greatly by using a first order approximation 10^−^*^x^* = 1 −*x*ln(10), which is valid for x<<1, and obtain the following approximate relation for Eq. (5).

(6) $$\begin{array}{l} 2.303(A-A_{buf})=a_{s}+a_{m}-a_{sp}\\ A-A_{buf}\leq 0.2 \end{array}$$

**Fig. 3:**
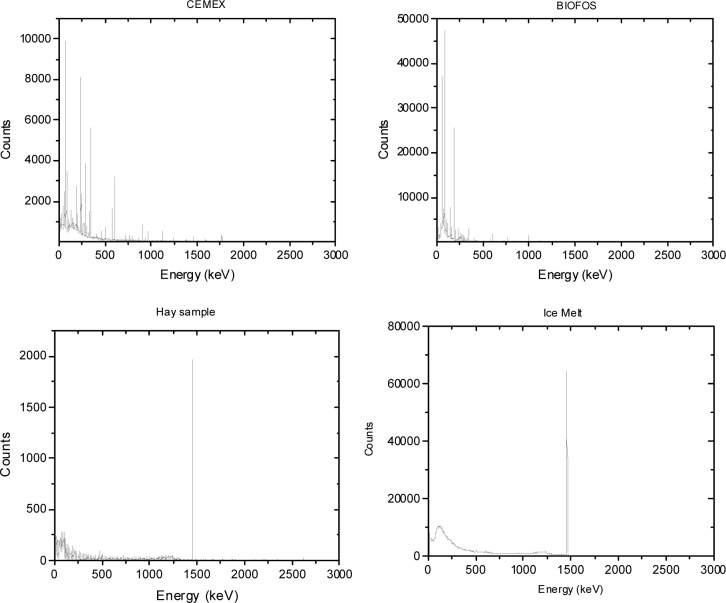
Model calculations of Mie scattering and absorption cross sections. The long dashed trace labeled σ_s_ shows the calculated total scattering cross section for microspheres with a diameter of 3.1 µm and an index of refraction of 1.59 + 0.007i. The short dashed trace labeled σ_sp_ shows the partial scattering cross section for scattered photons entering the instrument acceptance aperture. The solid trace σ_s_- σ_sp_ shows the expected instrument response, and the trace labeled σ_abs_ shows the expected molecular absorption cross section.

Equation (6) can be used to describe the measured absorbance when a cuvette is placed in any of the three holders. (It is assumed that the buffer is also measured for each holder). To a good approximation, *a_sp_* ≈ 0 for holder 1 and ${a^{\prime }}_{sp}\approx a_{s}$ for holder 3. The value of for holder 2 has to be calculated using Eq. (2) and the known concentration of microspheres. Figure 3 shows results of a calculation using Eq. (2) and Eq. (6) for polystyrene (PS) microspheres with diameter of 3.1µm and an index or refraction of 1.59 + 0.007i. The buffer index of refraction was set to 1.334 (water). The solid trace in Fig. 3, labeled *σ*_s_ - *σ*_sp_, is the predicted instrument response. The trace with short dashes gives *σ*_sp_ for an instrument with an assumed acceptance angle of 1.7°. The absorption cross section, *σ*_abs_, is small and is neglected in the description of the instrument response. In order to compare the calculated cross section with the data, the values of the cross sections have to be multiplied by a concentration, a unit conversion factor, and 1/2.303 to obtain the total response. Assuming the validity of Eq. (6) and a cuvette path length of 1 cm, the relation between the measured absorbance and the calculated cross section is given by Eq. (7).

(7) $$\begin{array}{l} 2.303(A-A_{buf})=N\cdot 10^{6}{\text{cm}}^{-3}\cdot \sigma \cdot 10^{-12}m^{2}\cdot 10^{4}\frac{{\text{cm}}^{2}}{m^{2}}\cdot 1\text{cm}\\ \;=N\cdot \sigma \cdot 0.01 \end{array}$$

As an example, if measurements were performed on a cuvette with a suspension of microspheres with a nominal concentration of 2.0 10^6^ cm^−3^, the trace labeled *σ*_s_ - *σ*_sp_ in Fig. 3 would be multiplied by a concentration 2.0 and 0.01/2.303 to give a predicted absorbance of about 0.13. In Eq. (7) and in the following, the calculated cross sections are presented in units of µm^2^, and the microsphere concentrations in units of 10^6^ cm^−3^.

## 3. Measurement of the Concentration of Microspheres

**Fig. 4:**
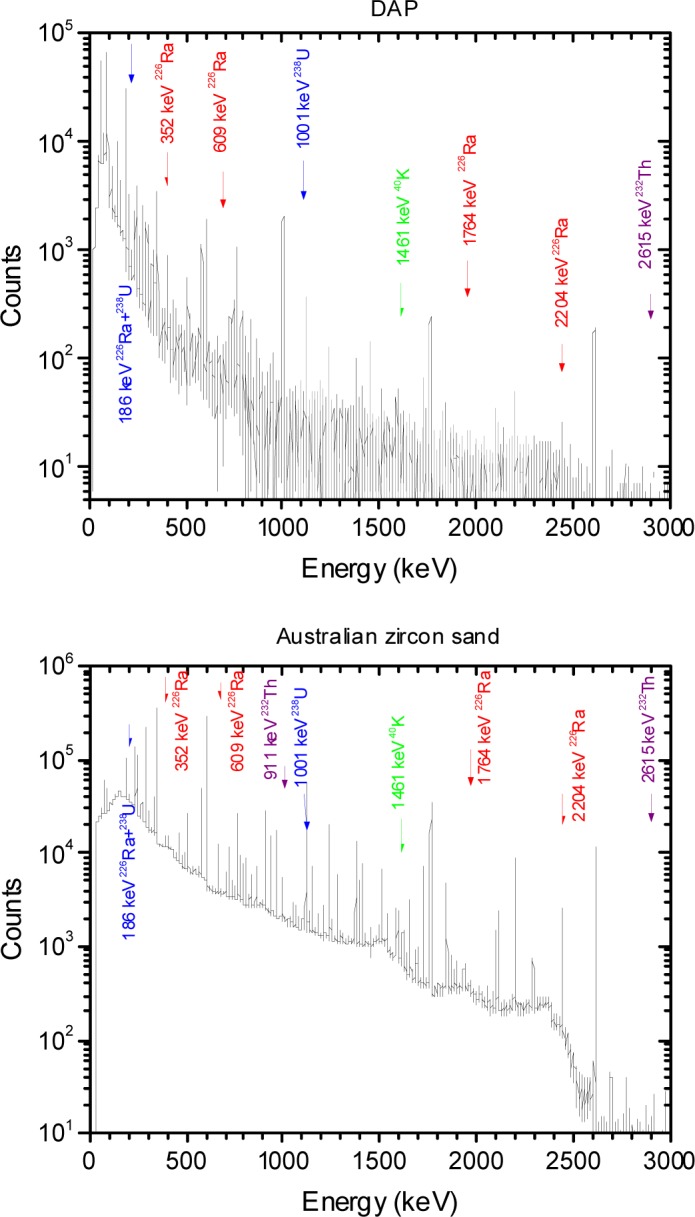
A scatter plot of events detected by the ARIA II flow cytometer during the passage of a suspension containing both TruCount microspheres and test microspheres. SSC stands for the side scattering signal and FSC stands for the forward scattering signal. Gate 1 and Gate 2 show the events associated with TruCount and test microspheres respectively. The density of dots is not representative of the density of events. The table gives the number of events associated with each gate.

The concentration of microspheres with a diameter of 3.1 µm was measured using an Aria II^1^ flow cytometer and a count standard provided by Beckman Dickinson Corporation. The count standard, with a trade name TruCount, was a vial containing a specified number of lyophilized microspheres. The TruCount standards were re suspended in 0.5 mL of the test microsphere suspension used in the absorbance measurements. The combined microsphere suspension was passed through a flow cytometer and the relative number of events associated with TruCount and test microspheres was recorded. The TruCount microspheres were smaller than the test microspheres so that the forward and side scattering signals provided by the flow cytometer could easily distinguish between the two types of microspheres. Figure 4 shows the dot plot of scattering events recorded by the flow cytometer. SSC and FSC stand for side scattering channel and forward scattering channel respectively. Gate 1 in Fig. 4 contains the events associated with the TruCount microspheres, and Gate 2 contains the events associated with the test (3.1 µm diameter) microspheres. The associations were validated by passing the separate microsphere suspensions through the flow cytometer. The concentration of the test microspheres was obtained by assuming that the relative number of the two types of microspheres counted by the flow cytometer was the same as the relative number of the two types of microspheres in the vial. The assumption leads to the relation *N_s_* = *C_s_ C_T_ N_T_* 0.5⋅ where *N_s_* is the concentration of microspheres, *N_T_* is the number of TruCount microspheres (given as 51085), *C_s_* is the number of test microspheres recorded by the flow cytometer (events in Gate 2), and *C_T_* is the number of TruCount microspheres recorded by the flow cytometer (events in Gate 1). Using the numbers of events of each type, the concentration of the test microspheres was estimated at 1.04*10^6^ cm^−3^. A detailed examination of the distribution of the events in Gate 2 shows that the standard deviation of the distribution is about 2.6 % of the mean forward scattering signal. This indicates that the microspheres are reasonably uniform and a calculation which assumes a unique microsphere diameter is reasonable.

## 4. Measurement of Absorbance in Scattering Suspensions

Measurements were performed on microspheres suspended in an aqueous PBS buffer or deionized water. The PerkinElmer Lambda 850 spectrophotometer was equipped with a 150 mm integrating sphere detector. The spectrometer was scanned from 210 nm to 800 nm in steps of 1 nm with an integration time of 0.52 s. The incident light monochromator was set to a resolution of 2 nm. A measurement sequence consisted of buffer measurements in holder 1 and 3 followed by the measurement of the microsphere suspension in holders 1 and 3. There was no stirring in holder 3, however effects due to settling were not observed during the measurement time (5 minutes) in holder 3.

**Fig. 5:**
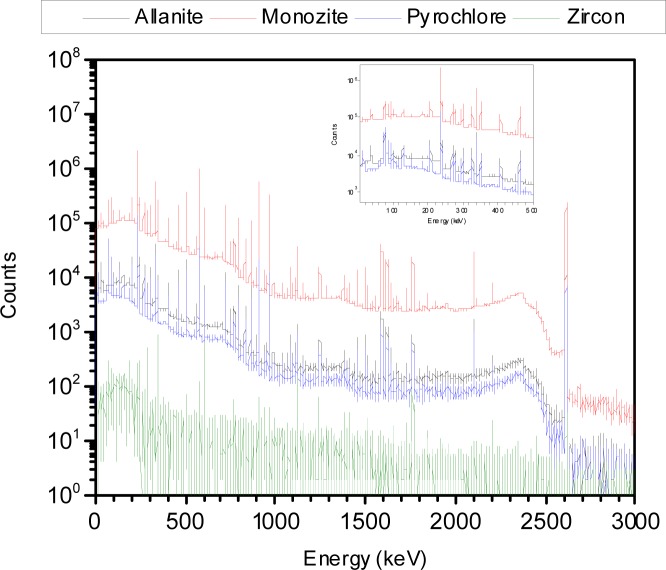
(a) The labeled traces show the measured absorbance from a cuvette filled with PBS buffer and placed in the three holders H1, H2, and H3. Trace H2 is slightly smaller than trace H1 due to the additional reflection caused by the cuvette placed in front of the IS entrance port. The measured absorbance at wavelengths close to 200 nm is due to molecular absorption by the buffer. For wavelengths greater than 250 nm the absorbance recorded with PBS was identical to that recorded for distilled water. (b) The labeled traces show the measured absorbance from a cuvette filled with microspheres suspended in PBS buffer and places in the three holders H1, H2, and H3. In all cases the buffer contribution was subtracted. The three traces are dramatically different reflecting the different instrument acceptance angles as discussed in the text. The measured absorbance in H3 is due to molecular absorption for wavelengths less than 300 nm, and backward scattered light escaping through the entrance aperture of the IS detector for wavelengths greater than 300 nm.

**Fig. 6:**
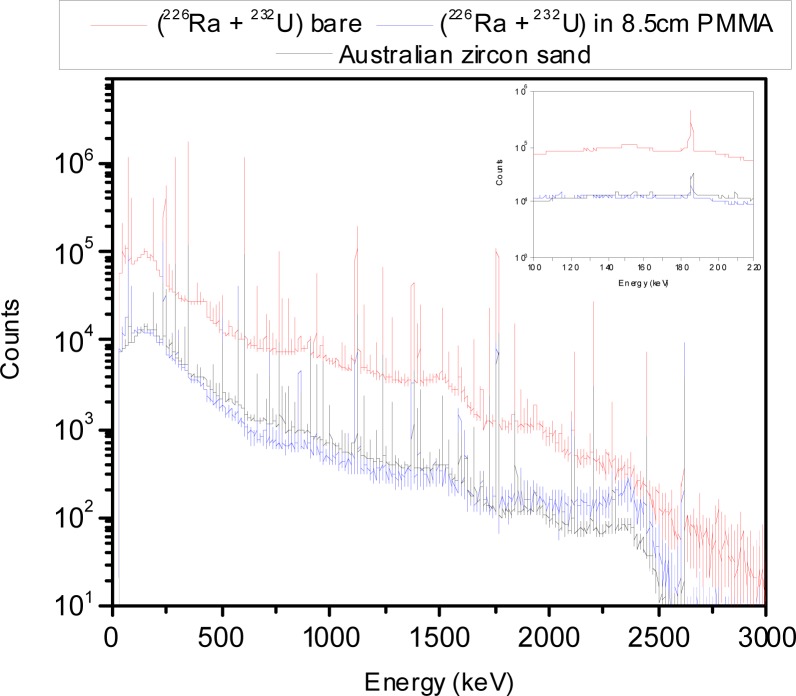
The dotted traces show the measured apparent scattering cross section of microspheres of diameter 1.5 µm, 2.6 µm, 3.0 µm, and 4.5 µm. The traces were obtained by subtracting the molecular absorbance (trace H3 in Fig 5b) from the total absorbance (trace H1 in Fig 5b) and dividing by the parameter c described in the text. The solid traces in Fig 6 show the calculated scattering cross section for microspheres using parameters shown in Table 1. The difference between the dotted and solid traces was less than 3 % except for wavelengths near 300 nm. In the case of the 4.5 µm microspheres the correspondence between the data and the fit was unsatisfactory for wavelengths below 450 nm.

The microsphere suspensions were sufficiently dilute so that the absorbance was less than 0.2, and the simplified analysis given by Eq. (6) and Eq. (7) could be used to model the data. The solid trace (H1) in Fig. 5a shows the measured PBS buffer absorbance in holder 1, the dotted trace (H2) and the dashed trace (H3) show the measured buffer absorbance in holders 2 and 3 respectively. The trace H2 in Fig. 5a is slightly smaller than the solid trace H1. The reduction is due to the change in IS response due to the placement of a reflecting surface in front of the entrance port of the IS detector. The dashed trace H3 in Fig. 5a shows a small residual absorbance which is most likely due to incident light reflected from the front surface of the cuvette and escaping through the entrance port of the IS. The three buffer measurements shown in Fig. 5a were subtracted from the three corresponding microsphere suspension measurements to yield the traces shown in Fig. 5b. The solid trace, H1, and the dotted trace, H2, in Fig. 5b show the microsphere absorbance measured in holders 1 and 2 respectively. According to Eq. (6), the response is composed of contributions from scattering and molecular absorption. The dashed trace (H3) in Fig. 5b shows the absorbance measured in holder 3 which according to Eq. (6) is primarily due to molecular absorption. Thus trace H3 in Fig. 5b can be used to estimate the molecular absorption *a_m_* which appears to be significant only for wavelengths less than 300 nm. A closer examination of trace H3 for wavelengths greater than 300 nm suggests that the small observed absorbance in trace H3 is due to a large degree to backward scattered light escaping the IS through the input port. Assuming that the molecular absorption is negligible for wavelengths greater than 300 nm, trace H2 provides another estimate of *a_m_* given by the component of the measured absorbance above a constant background due to scattering. The estimate of *a_m_* from trace H3 is influenced by the presence of fluorescence, and the estimate of *a_m_* from trace H2 assumes a constant scattering background. However the two estimates are consistent and the response recorded in H3 was used to subtract the molecular absorption contribution from trace H1 in Fig. 5b. The resulting trace, after division by the concentration factor (see Eq. (7)), is shown by the dotted traces in Fig. 6. The solid traces in Fig. 6 show the results of fitting the model to the data and are the subject of the following section.

## 5. Analysis of the Absorbance Due to Scattering

The dotted traces in Fig. 6 show the apparent scattering cross section for PS microspheres with a diameter of 1.5 µm, 2.6 µm, 3.0 µm, and 4.5 µm suspended in distilled water (DI). The solid traces in Fig. 6 show the best fit to the model based on Lorenz-Mie calculation. The Mie calculations were performed using MatLab with Maetzler code for Lorenz-Mie scattering. The fit shown in Fig. 6 resulted from the minimization of residuals defined in Eq. (8).

(8) $$\begin{array}{l} \text{Residuals}=\sum_{\lambda }(\frac{A_{1}-A_{3}}{c}-\text{Mie}(d,n)+\int_{0}^{\Delta }\frac{d\sigma }{d\theta }(d,n,\theta )d\theta {)}^{2}\\ c=N\cdot 0.01/2.303\\ d=\text{diameter}\\ n=\text{index of refraction}\\ \Delta =\text{acceptance angle for holder}\;1 \end{array}$$

The quantities *A_1_* and *A_3_* stand for the measured absorbance in holders 1 and 3 respectively (with the buffer contributions subtracted). The quantity *Mie(d,n)* is the calculated total scattering cross section. The integral of the differential Mie scattering cross section was performed over the effective angle (symbol Δ in Eq. (8)) subtended by the instrument entrance aperture at the location of the cuvette in holder 1. Because of the finite detector aperture, some of the radiation scattered in the forward direction will enter the detector and reduce the absorbance. The purpose of the integral in Eq. (8) is to model this reduction. The acceptance angle was assumed to equal the acceptance angle determined for the optical system in air divided by the wavelength dependent index of refraction of water [9]. The index of refraction of water takes into account the refraction of light at the cuvette wall. Specifically:

(8a) $$\Delta =\frac{\Delta_{0}}{\left(1.3128+0.015762/\lambda -0.004382/\lambda^{2}+0.00146/\lambda^{3}\right)}$$

The symbol Δ_0_ is the detector acceptance angle for the system in air. (The values of the coefficients in the expansion of the index of refraction in Eq. (8a) were changed to accommodate the µm units of wavelength which were used in the Mie calculation.) Detailed calculations based on geometric optics [10] have shown that the refraction at the cuvette wall is the most important correction to the collection efficiency in luminescence measurements. For the purpose of characterizing the collection efficiency, scattered photons can be viewed as luminescence. The main difference is that while luminescence is emitted approximately equally in all directions, the scattered photons are predominantly emitted in the forward direction. The estimate of the collection efficiency in Eq. (8) assumes optimal collection from all illuminated regions in the cuvette; this assumption may lead to an overestimate of the collection efficiency.

The residuals in Eq. (8) were summed over a selected range of wavelengths with the lower bound always set to a wavelength larger than 300 nm. It was possible to obtain an excellent fit for wavelength region between 500 nm and 800 nm with a constant value of the index of refraction for the microspheres. For fitting below 500 nm, it was necessary to introduce a wavelength dependent index of refraction given by Eq. (9).

(9) $$n(\lambda )=(A+\frac{B}{\lambda^{2}}+\frac{C}{\lambda^{4}})$$

The imaginary part of the index of refraction was set to zero in accordance with observation of minimal absorption in holder 3 for wavelengths above 300 nm. The parameterization in Eq. (9) was based on observations in other materials which display an increased index of refraction at lower wavelengths. The medium (water) index of refraction was set to 1.334. The values of *n*(*λ*) obtained from Eq. (9) are not directly equal to the PS index of refraction. Some algebra and the dependence on wavelength of the index of refraction of water are needed to extract the true PS index of refraction as discussed below. The fit between the measured and calculated values shown in Fig. 6 was performed for wavelength range 300 nm to 800 nm. The values of the fit parameters which resulted in the best fits are shown in Table 1.

The fits in Fig. 6 are excellent for wavelengths greater than 450 nm, the differences between the data and calculated values are less than 3 %. For the smaller microspheres the fit is still very good down to 300 nm, however the fit for 4.5 µm microsphere becomes poor for wavelengths less than 450 nm. In all cases, the diameter extracted from the fit was very close to the diameter quoted by the manufacturer. The poorer fit for *λ* < 400 nm suggests a need for a better representation of the detector collection efficiency. The relatively small difference between the calculated and measured values in Fig. 6 indicates that the modeling described in this work has validity.

**Fig. 7:**
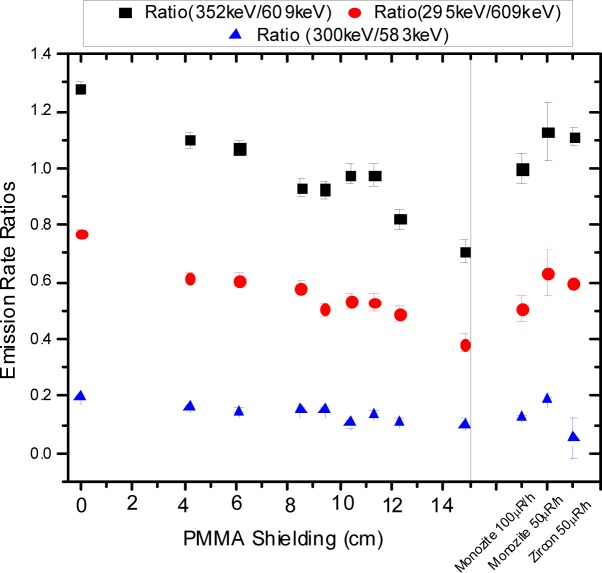
The dotted and solid traces are the same as in Fig 6. The dashed trace shows the calculated cross section with the detector acceptance angle set to zero. The dashed curve for the 1.5 µm data differs from the solid curve by at most 16 %. In contrast the difference for the 4.5µm microspheres averages to about 60 %. Clearly such a large difference is more than a correction and points to the need for an improved analysis of the instrument collection efficiency.

The parameters in Table 1 were used to calculate the expected response if the detector acceptance angle was vanishingly small. The dashed traces in Fig. 7 show the resulting calculation which can be compared with the dotted and solid traces reproduced from Fig. 6 for the 1.5 µm and 4.5 µm microspheres. For the case of 1.5 µm microspheres, the difference between the dashed and solid traces in Fig. 7 is less than 16 %. Thus the acceptance angle correction is relatively small. For the case of 4.5 µm microspheres, the difference between the dashed and solid traces is on the average about 60 % suggesting that the collection efficiency estimate is an integral part of the measurement and cannot be treated as a “correction”. The modeling of the measured response could be enhanced by developing a better estimate of the detector collection efficiency.

### 5.1 Estimate of Index of Refraction

The results from the fits to the microsphere data were used to estimate the PS index of refraction. Let *n_m_*(*λ*) correspond to the index of refraction obtained from the fit with the parameters discussed in Eq. (9). The index of refraction of PS can be obtained from the equality of the ratios given in Eq. (10).

(10) $$\frac{n_{m}}{1.334}=\frac{n_{PS}}{n_{W}}$$

Where *n_PS_* and *n_W_* are the true indexes of refraction of polystyrene and water respectively. The ratio *n_m_*/1.334 was used to fit the data because it simplified the algorithm. However if the fit is good, then the ratio *n_m_/*1.334 should be equivalent to the ratio *n_PS_/n_W_* as indicated in Eq. (10). If the assumption is made that the index of refraction of water is real, then Eq. (10) holds for both the real and imaginary parts of the PS index of refraction. Using the real part of the measured index of refraction given in Eq. (9) and the known water index of refraction [9], the polystyrene index of refraction, *n_PS_*, is given by Eq. (11).

(11) $$n_{PS}(\lambda )=\frac{A+B\cdot 10^{6}/\lambda^{2}+C\cdot 10^{12}/\lambda^{4}}{1.344}\cdot (1.3128+15.762/\lambda -4382/\lambda^{2}+1.1455\cdot 10^{6}/\lambda^{3})$$

The values of *A*, *B*, and *C* were obtained from the fits to the data from Eq. (9), the factors 10^6^ and 10^12^ multiplying *B* and *C* convert the coefficients to a wavelength scale in nanometers. The PS index was computed in accordance to Eq. (11) and the computed values were fitted to a power expansion of the form *c*_0_ + *c*_1_*λ*^−2^ + *c*_2_*λ*^−4^. Table 2 gives the values of the coefficients obtained for three microspheres with diameters 1.5 µm, 2.6 µm, and 3.0µm. The 5^th^ column in Table 2 gives the results of Ma [11] for measurements of the index of refraction of PS microsphere with a diameter of 1 µm.

The reasonably close results for the measurements on the three spheres give credence to the PS result which is valid above wavelengths of 300 nm. Ma’s results give less pronounced wavelength dependence. The values of the index of refraction at large wavelengths are identical to Ma’s value or the value given by Nikolov [12] for the index of refraction of bulk PS. The discrepancy in the wavelength dependence of the PS index of refraction is most likely due to the approximation in the estimate of the collection efficiency in Eq. (8). The parameters *B* and *C* in Eq. (9), and the collection efficiency affect the wavelength dependence of the calculated response, and are therefore coupled during the fitting process. Inaccuracies in the estimate of the collection efficiency would be offset by adjusting the values of the parameters *B* and *C*.

### 5.2 Estimate of Diameter and Concentration

The value of the microsphere diameter was obtained directly from the fit to the data. The diameters obtained for the three microspheres matched reasonably well the nominal values given by the supplier. It has been observed that the analysis of Lorenz-Mie scattering provides good estimates of particle diameters either from angular dependence measurements [13] or wavelength dependence measurements [14]. The structure present in the wavelength dependence of the scattering cross section provides a strong constrain to the microsphere diameter fit parameter.

The microsphere concentration, *N*, can in principle be obtained from the relation *N* = 230.3 · *c* where *c* is the fit parameter given in Eq. (8). The parameter *c* provides a scaling of the entire cross section without changing the shape of the wavelength dependence. If the initial guess of the parameters gives a reasonable representation of the data then the fit converges to unique values of *d*, *c*, and *n*. If the initial guess is too far off, then it is possible for the *c* and *d* parameters to become coupled during the fit giving unreasonable results. The value of the parameter c in row labeled “3.1 µm” in Table 1, gives a concentration of 2.35*10^6^ cm^−3^. This value can be compared with the estimated concentration 1.04*10^6^ cm^−3^ obtained using the flow cytometer and the BD count standard. The flow cytometer measurement has its own systematic uncertainties; however the difference between the flow cytometer measurement and the scattering measurement is outside any reasonable estimate of uncertainty. The concentration of the 3.1 µm microspheres was also estimated by using the concentration of the stock suspension provided by the manufacturer; the result was 1.67*10^6^ cm^−3^. In the case of 1.5 µm and 2.6 µm microspheres, the estimated concentration from the manufacturer’s values was about 7 *10^6^ cm^−3^ while the estimates from parameter *c* were about 11*10^6^ cm^−3^. The values of concentration obtained from the fit parameter *c* exceed the concentration values obtained from the flow cytometer or from the manufacturer by a factor of approximately 1.6. Clearly there is a systematic bias between the different methods for measuring concentration. In the case of scattering measurements, the collection efficiency estimate influences the overall magnitude of the calculated cross section and thus has a significant effect on the value of the parameter *c*. A reduction in the collection efficiency of 60 % would reconcile the concentrations obtained from the parameter *c* with those obtained from flow cytometer and manufacturer’s estimates. Such a reduction in the collection efficiency is reasonable. In order to obtain a more accurate estimate of the concentration, the collection efficiency estimate in Eq. (8) has to be modified to include the decrease in collection efficiency from the illuminated regions in the cuvette which are not located at the image of the detector aperture inside the cuvette. In addition, an estimate is needed of the “effective transmittance” of the scattered light for the cuvette placed inside the IS detector.

## 6. Conclusion

A careful analysis of the measurement process in a spectrometer with an integrating sphere (IS) detector led to a procedure for separating the measured absorbance into components due to scattering and molecular absorption. The analysis hinged on the interpretation of absorbance measured for a cuvette placed at two different holders in the spectrometer. Holder 1 corresponds to the normal position and holder 3 locates the cuvette inside the IS detector. Equation (7) and Eq. (8) give the relationship between the measured responses (*A*_1_, *A*_3_) and the analyte properties (*a_s_*, *a_m_*,*a_sp_*). Approximate forms of the measurement model equations were used to analyze the absorbance from polystyrene spheres suspended in phosphate buffer saline (PBS) and distilled water (DI). The results suggest that the model is valid and that it is indeed possible to separate the two contributions. The two quantities, *a_s_*, *a_m_*, are independent characteristics of the microsphere suspension. The quantity *a_m_* gives information about the electronic states of the absorbing styrene molecules, while the quantity *a_s_* provides information about the microsphere diameter, the concentration, and the wavelength dependence of the index of refraction of the material inside the microsphere. Further work is needed to clarify the systematic uncertainties inherent in the measurement model. The most significant of these uncertainties is in the estimation of the partial cross section *a_sp_* which depends on both the collection efficiency of the detector and the angular distribution of the scattered radiation. For wavelengths greater than 300 nm, the measurement and analysis of the scattering cross section provides estimates of the microsphere diameter, the microsphere concentration, and the wavelength dependence of the index of refraction of the microsphere material. It is likely that the poor fit between the data and calculation at wavelengths approaching 300 nm and large microsphere diameters is due to approximations in the estimate of the collection efficiency of the forward scattered light. More accurate estimates of the collection efficiency would provide a robust technique to measure the properties of suspensions of homogeneous microspheres.

## Figures and Tables

**Table 1:** Parameters of fits in Fig. 6

Bead	A	B	C	d, µm	c	Δ_0_, deg
1.5 µm	1.576	0.00130	0.000792	1.50	0.0486	5.24
2.6 µm	1.588	0.000539	0.000667	2.45	0.0440	3.81
3.0 µm	1.583	0.000477	0.000729	3.01	0.0100	3.85
3.1 µm	1.585	0.000578	0.000631	3.06	0.0102	3.93
4.5 µm	1.566	0.000272	0.00195	4.47	0.0043	4.03

**Table 2:** Comparison of the wavelength dependence of PS microsphere index of refraction

	1.5 µm	2.6 µm	3.0 µm	Ma [11]
c_0_	1.567	1.579	1.574	1.573
c_1_	3061	2503	2355	3108
c_2_	9.64*10^8^	8.22*10^8^	8.91*10^8^	3.48*10^8^

## Footnotes

1 Certain commercial equipment, instruments, and materials are identified in this paper to foster understanding. Such identification does not imply recommendation or endorsement by the National Institute of Standards and Technology.

## References

[1] Fecht I, Johnson M (1999). Non-contact, scattering-independent water absorption measurement using a falling stream and integrating sphere. Measurement Science and Technology.

[2] Babin M, Stramski D (2002). Light absorption by aquatic particles in the near-infrared spectral region. Limnology and Oceanography.

[3] Phillips DT, Wyatt PJ, Berkman RM (1970). Measurement of the Lorenz-Mie Scattering of a Single Particle: Polystyrene Latex. Journal of Colloid and Interface Science.

[4] Bohren CF, Huffman DR (1983). Absorption and Scattering of Light by Small Particles.

[5] He GS, Qin H-Y, Zheng Q (2009). Rayleigh, Mie, Tyndal scattering of polystyrene microspheres in water: Wavelength, size, and angle dependence. Journal of Applied Physics.

[6] Gaigalas AK, He H-J, Wang L (2009). Measurement of Absorption and Scattering with an Integrating Sphere Detector: Application to Microalgae. Journal of Research of the National Institute of Standards and Technology.

[7] vd Hulst HC (1957). Light Scattering by Small Particles.

[8] Maetzler C (2002). MATLAB Functions for Mie Scattering and Absorption.

[9] Huibers PDT (1997). Model for the wavelength dependence of the index of refraction of water. Applied Optics.

[10] Ediger MD, Moog RS, Boxer SG, Fayer MD (1982). On the refractive index correction in luminescence spectroscopy. Chemical Physics Letters.

[11] Ma X, Lu JQ, Brock RS, Jacobs KM, Yang P, Hu X-H (2003). Determination of complex refractive index of polystyrene microspheres from 370 to 1610 nm. Physics in Medicine and Biology.

[12] Nikolov ID, Ivanov CD (2000). Optical plastic refractive measurements in the visible and the near-infrared regions. Applied Optics.

[13] Weiner I, Rust M, Donnelly TD (2001). Particle Size Determination: An Undergraduate Lab in Mie Scattering. American Journal of Physics.

[14] Chamberlin D, Trutna R (2008). Physics of Particle Size Spectrophotometry.

